# HALF OF OLDER ADULTS HOSPITALIZED WITH COVID-19 EXPERIENCE SYMPTOMS ONE YEAR LATER

**DOI:** 10.1093/geroni/igac059.2982

**Published:** 2022-12-20

**Authors:** Aleda Leis, Sydney Fine, E J McSpadden, Carrie Karvonen-Gutierriez, Emily Nichols, Lois Lamerato, Anurag N Malani, Emily Martin

**Affiliations:** University of Michigan, Ann Arbor, Michigan, United States; University of Michigan School of Public Health, Ann Arbor, Michigan, United States; University of Michigan, Ann Arbor, Michigan, United States; University of Michigan, Ann Arbor, Michigan, United States; UM Michigan Center for Respiratory Research and Response, Ann Arbor, Michigan, United States; Henry Ford Health, Detroit, Michigan, United States; St. Joseph Mercy Hospital, Ypsilanti, Michigan, United States; University of Michigan School of Public Health, Ann Arbor, Michigan, United States

## Abstract

Older adults hospitalized with severe COVID-19 are at higher risk of experiencing serious in-hospital outcomes and long-term health consequences following discharge. Declines in health and functional ability post-hospitalization are important infection-related outcomes. This study’s aim was to examine functional recovery one year following COVID-19 hospitalization. Twenty-one adults ≥60 years of age hospitalized with confirmed COVID-19 infection between 3/2020–5/2020 in Southeast Michigan completed a survey 9–15 months post-discharge including items from the Fried Frailty score, Short Form 36 Physical Assessment, PROMIS Dyspnea Scale, and the World Health Organization Disability Assessment Schedule. Mean age at hospital admission was 69 (standard deviation 7). Half of participants (52%) indicated they had too little energy to do the things they wanted to do, 52% (n=11) indicated moderate to severe shortness of breath when walking up two flights of stairs, and 43% (n=9) indicated they were limited a lot in walking several blocks. Additionally, 57% (n=12) indicated they were severely or extremely emotionally affected by their health due to their COVID-19 infection. Results were similar in only those ≥70 years (n=7). Our survey indicates that half of patients hospitalized with severe COVID-19 from the first infection wave in Southeast Michigan are significantly affected up to a year or more after their initial infection, and may benefit from long-term outpatient care. More research is needed to inform development of effective treatments for the long-term emotional and physical impacts of severe COVID-19.

